# LncRNA GAS5 regulates redox balance and dysregulates the cell cycle and apoptosis in malignant melanoma cells

**DOI:** 10.1007/s00432-018-2820-4

**Published:** 2018-12-19

**Authors:** Long Chen, Huixin Yang, Zihan Yi, Lu Jiang, Yuqian Li, Qiaoqiao Han, Yuye Yang, Qiao Zhang, Zhe Yang, Yingmin Kuang, Yuechun Zhu

**Affiliations:** 1 Department of Biochemistry and Molecular Biology, Kunming Medical University, No. 1168 Yuhua Road, Chenggong District, Kunming, 650500 Yunnan China; 2 PET-CT Center, Yunnan Tumor Hospital, Third Affiliated Hospital of Kunming Medical University, Kunming, China; 3 Department of Pathology, The First Affiliated Hospital of Kunming Medical University, Kunming, China; 4 Department of Organ Transplantation, First Affiliated Hospital of Kunming Medical University, No. 295 Xichang Road, Wuhua District, Kunming, 650032 Yunnan China

**Keywords:** Reactive oxygen species, Cell cycle, Apoptosis, LncRNA, GAS5, Malignant melanoma

## Abstract

**Purpose:**

Clinical outcomes for advanced malignant melanoma (MM) are often poor due to tumor invasiveness, metastasis, recurrence, and multidrug resistance.

**Methods:**

We investigated whether apoptosis, cell cycle regulation, oxidative status, and redox balance were altered by changes in the expression of the long noncoding RNA, growth arrest-specific transcript 5 (GAS5), in MM cells.

**Results:**

Analysis of clinical samples from MM patients showed that the rate of reduced GAS5 expression, relative to that in adjacent noncancerous tissues, was significantly lower for tumors from patients with advanced disease (76.6%, *P* < 0.001), as evidenced by larger tumor size, higher TNM stage, and higher incidences of ulceration and metastasis (*P* < 0.001 for all). Cell culture experiments showed that siRNA-mediated knockdown of GAS5 increased the viability of A375-GAS5si cells. Flow cytometry and western blotting showed that GAS5 knockdown increased MM cell proliferation by inducing G1/S cell cycle progression through increases in Cyclin D1, CDK4, and p27 expression (*P* < 0.05 for all) and by inhibiting apoptosis through an increase in Bcl-2 expression (*P* < 0.001). Knockdown of GAS5 also increased levels of superoxide anion (*P* < 0.01), NADP^+^(*P* < 0.001), and oxidized glutathiones (*P* < 0.01) through increases in NOX4 expression (*P* < 0.001), G6PD expression (*P* < 0.01), and NOX activity (*P* < 0.05), and RNA co-immunoprecipitation showed that GAS5 induced these changes through a physical interaction between GAS5 and the G6PD protein.

**Conclusions:**

Our findings show GAS5 contributes to regulation of the apoptosis, cell cycle, homeostasis of reactive oxygen species, and redox balance in MM cells, and suggest that reduced GAS5 expression contributes to disease progression in MM patients.

**Electronic supplementary material:**

The online version of this article (10.1007/s00432-018-2820-4) contains supplementary material, which is available to authorized users.

## Introduction

The incidence of malignant melanoma (MM) has increased in recent decades, especially in economically developed regions (Siegel et al. 2017; Torre et al. 2015). Surgery alone is sufficient to achieve long-term survival in approximately 80% of patients who are diagnosed early (Fruehauf and Trapp 2008; Gidanian et al. 2008). However, clinical outcomes for advanced MM are often poor due to tumor invasiveness, distant metastasis, recurrence, and multidrug resistance (Maverakis et al. 2015; Niezgoda et al. 2015). Despite the availability of a number of treatment options, including surgery, targeted chemotherapy, immunotherapy, and radiotherapy, the 5-year survival rate for stage-IV MM remains < 18% (Fruehauf and Trapp 2008; Maverakis et al. 2015), and recently developed therapeutic agents for MM have not demonstrated significant improvement in long-term survival (Enewold et al. 2017; Garbe et al. 2011; Maverakis et al. 2015). Efforts to develop more effective treatment strategies for MM require the identification of biochemical mechanisms crucial to MM progression.

Elevated levels of reactive oxygen species (ROS) occur in rapidly proliferating tumor cells as a result of increased ATP requirements, hypoxia, and oncogene-mediated signaling (Gill et al. 2016; Szatrowski and Nathan 1991). Melanosome exposure to UV-radiation further contributes to ROS production in MM cells (Gidanian et al. 2008; Wittgen and van Kempen 2007). Certain ROS can activate a variety of signaling pathways regulating the viability, proliferation, and the metabolic adaptation of cells (Hambright et al. 2015; Venza et al. 2015), such as the stimulation of G_1_→S progression by H_2_O_2_ (Coats et al. 1996; Rivard et al. 1996). Radiotherapy and some anticancer drugs stimulate apoptosis by increasing ROS levels beyond tolerable thresholds (Gill et al. 2016; Gorrini et al. 2013), but the mechanisms underlying the recalcitrance of MM to such treatments remain unclear (Cesi et al. 2017; Gorrini et al. 2013). The upregulation of oxygen-scavenging systems may contribute to resistance to anticancer treatments and subsequent disease recurrence (Diehn et al. 2009). Although the ROS-induced stimulation of autophagy can reduce oxidative stress and promote tumor cell survival under certain conditions (Poillet-Perez et al. 2015), a recent study showed that the inhibition of mitochondrial complex I resulted in increased ROS levels and mitophagy-dependent ferroptosis in BRAF^V600E^ MM cells (Basit et al. 2017). Therefore, a better understanding of the regulation of ROS production in MM cells and the identification of its links to apoptotic and non-apoptotic cell death mechanisms are needed.

Certain long noncoding RNA (lncRNA) have been shown to be involved in the tumorigenesis and metastasis of various types of cancer (Anastasiadou et al. 2017; Esteller 2011; Qiu et al. 2013) through interactions with protein and microRNA (miRNA) targets (Tsai et al. 2011). Examples of these include the lncRNA HOTAIR-induced dysregulation of chromatin formation and histone methylation in breast cancer (Gupta et al. 2010), the interaction of lncRNA CCAT1 and miRNA let-7 in the progression of hepatocellular carcinoma (Deng et al. 2015), and the lncRNA HEIH-mediated stimulation of MM cell proliferation, migration, and invasion. The lncRNA, growth arrest-specific transcript 5 (GAS5), has been implicated in dysregulation of the cell cycle and apoptosis in prostate cancer (Romanuik et al. 2010), breast cancer (Zhang et al. 2013b), and non-small cell lung cancer (Shi et al. 2015), and differential GAS5 expression was associated with poor clinical outcome and reduced survival for patients with glioblastoma multiforme (Zhang et al. 2013a), colorectal cancer (Yin et al. 2014), and cervical cancer (Cao et al. 2014).

Recent studies have reported reduced GAS5 expression in clinical MM samples, relative to control tissues (Wang et al. 2017), and have shown that reduced GAS5 expression inhibited apoptosis in the MM cell line, A375 (Wang et al. 2017). In our previous studies, GAS5 expression in A375 cells downregulated the expression of the matrix metalloproteinases (MMPs) gelatinase A (MMP2) and gelatinase B (MMP9), inhibited the migration of A375 cells, and reduced tumor volume in a mouse xenograft model of MM (Chen et al. 2016a, b). Given that ROS balance represents a potential therapeutic target for MM treatment (Wittgen and van Kempen 2007), our current study focused on determining whether apoptotic signaling pathways, cell cycle regulation, ROS homeostasis, and redox balance in MM cells were altered in response by changes in the level of GAS5 expression. Our results showed that GAS5 physically interacts with glucose-6-phosphate dehydrogenase (G6PD), and that reduced GAS5 expression inhibits apoptosis, induces G1/S progression, and alters intracellular ROS levels and redox balance.

## Materials and methods

### Clinical tissue specimens

Samples of MM tumors (≥ 2-cm surgical margins) and adjacent noncancerous tissues were obtained from 47 patients who underwent surgery between 2014 and 2016 at Yunnan Cancer Hospital in Kunming, China. Written informed consent was obtained from all of the patients prior the analysis of the clinical samples. Our study was approved by the Ethics Committee of Kunming Medical University. The histological diagnosis of MM was based on criteria established by the World Health Organization (WHO). Tissue samples were immediately frozen in liquid nitrogen, and stored at − 80 °C.

### Cell lines and cell culture methods

The A375 and SK-Mel-28 human MM cell lines were purchased from ATCC (Manassas, VA, USA). The A375-GAS5si and SK-Mel-110-GAS5over cell lines were generated in our previous study (Chen et al. 2016b). All cell lines were cultured at 37 °C in 5% CO_2_ in Dulbecco modified Eagle medium (DMEM, Life Technologies, Carlsbad, CA, USA) supplemented with 5% fetal bovine serum (FBS, Life Technologies), 100 U/mL penicillin, and 100 µg/mL streptomycin without exceeding a density of 0.5 × 10^6^ cells/mL. In rescue experiments, SK-Mel-110-GAS5 over cells were transfected with pSIREN-RetroQ-ZsGreen1-GAS5shRNA plasmid, as previously described (Chen et al. 2016b), and A375-GAS5si cells were transduced with a lentivirus expression system that was generated using the pLVX-IRES-ZsGreen1-GAS5 vector, as described previously (Chen et al. 2016a).

### Quantification of mRNA expression

Quantitative reverse transcription and real-time polymerase chain reaction (qRT-PCR) for the quantification of GAS5, β-actin, and GAPDH expression was performed as described previously (Chen et al. 2016b). Target mRNA levels were normalized to control mRNA levels, and relative gene expression was calculated using the ${2^{ - \Delta \Delta {C_{\text{t}}}}}$ method (Livak and Schmittgen 2001). Blank controls with no cDNA templates were included, and specificity was confirmed by melting curve analysis and gel electrophoresis. Results are expressed as mean ± SD from three-independent experiments.

### Cell viability assay

The viability of cultured MM cells was assessed by 3-(4,5-dimethylthiazol-2-yl)-2,5-diphenyltetrazolium bromide (MTT) assay using the Roche Cell Proliferation Kit I (Sigma-Aldrich). Cells were seeded in 96-well plates at a density of 2 × 10^3^ cells/well, and incubated for 12, 24, 36, 48, and 72 h in DMEM containing 10% FBS. The MTT solution was added to a final concentration 0.5 mg/mL, and the cells were incubated for 4 h before the formazan product was measured based on absorbance at 450 nm.

### Fluorescence in situ hybridization (FISH)

FISH staining of human GAS5 mRNA was performed as described previously (Raj et al. 2008) with modification. The probe was prepared by carboxy-tetramethylrhodamine end-labeling (5′-TAMRA-CAGGAGCAGAACCATTAAGCTGGTCCAGGCAAGT-TAMRA-3′). Fixed cells in suspension were washed with 0.1% Triton in 1× PBS, and adhered to poly-lysine-coated slides for 24 h. Slides were washed in 1× PBS, and fixed in 4% paraformaldehyde before permeabilization with 0.2 M HCl. Following a 70%, 85%, and 100% ethanol series, fluorescent probe hybridization was performed at 37 °C overnight. After three 5-min washings with 50% formamide in 2× SSC at room temperature, the slides were counterstained with DAPI. Confocal microscopy images were recorded, and image analysis was performed in Matlab.

### Western blotting

Total protein concentration was determined using BCA reagent (Thermo Fisher Scientific, Waltham, MA, USA). SDS-PAGE was performed using an 8% acrylamide gel. Western blotting was performed as described previously (Chen et al. 2016b). Rabbit monoclonal anti-G6PD, rabbit polyclonal anti-β-actin, and mouse monoclonal anti-NADPH oxidase 4 (NOX4) antibodies were purchased from Abcam (Cambridge, MA, USA). The rabbit polyclonal anti-Caspase 3, anti-Bcl-2, mouse monoclonal anti-Cyclin D1, mouse monoclonal anti-p21, mouse monoclonal anti-p27, mouse monoclonal anti-cyclin dependent kinase-4 (CDK4), and mouse monoclonal anti-GAPDH antibodies were purchased from Santa Cruz Biotechnology (Dallas, TX, USA). Horseradish peroxidase-conjugated secondary antibodies were purchased from Sigma-Aldrich. Band densities were quantified using the ImageJ 1.46r software (NIH, USA). Results are expressed as the ratio of target band density to that of β-actin (loading control). Changes in expression are reported as percentage of the control, or as fold difference, as defined by FD = (*B*/*A*) − 1, where *A* is the reference value of the dependent variable and *B* is the value of the dependent variable after independent variable manipulation. For altered ROS conditions, cells were exposed to 50 µM H_2_O_2_ or 100 µM *N*-acetyl-cysteine (NAC) for 45 min.

### Flow cytometric analyses of cell cycle distribution and apoptosis

For cell cycle distribution analysis, 5 × 10^5^ cells were cultured in 60 mm dishes overnight at 37 °C. Cells were harvested by trypsinization, washed twice with 1× PBS, fixed in 70% ethanol overnight at 4 °C, and incubated with 0.5 mL propidium iodide (PI) in Triton X-100 staining solution with Ribonuclease A for 30 min. Cell sorting was performed using a FACScan flow cytometer (BD Biosciences, San Jose, CA, USA), and percentages of cells in the G1, S and G2/M phases were calculated using CellQuest Pro software version 5.1 (BD Biosciences). Apoptosis of MM cells was assessed based on Annexin V and 7AAD staining, as described previously (Adan et al. 2017). Cells were seeded in 25 cm^2^ flasks. Trypsinized cells were resuspended in 1 mL of 1× binding buffer (BB), and centrifuged at 300×*g* for 10 min. After supernatant removal, the cells were resuspended in 100 µL BB, followed by the addition of 5 µL Annexin V-APC and 7AAD-FITC (Invitrogen, Carlsbad, CA, USA) and incubation for 15 min at room temperature in the dark. After washing with 1 mL BB, cells were collected by centrifugation at 300×*g* for 10 min. After supernatant removal, cells were resuspended in 500 µL BB. Immediately prior to analysis, samples were combined with 10 µL PI (20 µg/mL; Sigma-Aldrich, St. Louis, MO, USA), and mixed gently. For each sample, at least 10,000 events were recorded and analyzed using a Cytomics FC500 flow cytometer with CXP software (Beckman Coulter, Fullerton, CA, USA). Percent apoptosis was calculated using Cyflogic 1.2.1 software (CyFlo, Turku, Finland). Necrotic (dead) cells are 7AAD-positive and Annexin V-negative, and are represented in the upper-left quadrant of the monochrome density plots. Non-viable (late) apoptotic cells are positive for both Annexin V and 7AAD, and are represented in the upper-right quadrant. Viable (early) apoptotic cells are 7AAD-negative and Annexin V-positive, and are represented in the lower-right quadrant. Viable non-apoptotic cells are negative for both Annexin V and 7AAD, and are represented in the lower-left quadrant.

### Quantification of ROS level in vivo

In vivo detection of ROS was performed as previously described (Anderica-Romero et al. 2016). Cells were incubated in 20 µM dihydroethidium (DHE) in DMEM without phenol red for 30 min at 37 °C, and examined using a fluorescence microscope (excitation 510–560 nm; emission 590 nm) for preliminary ethidium detection. The ROS level was quantified by a FACScan flow cytometer (BD Biosciences). Red fluorescence was evaluated at 590–700 nm (excitation 488 nm; FL-2 channel emission 525–625 nm). Apoptotic cells were excluded by DAPI counter staining. Data are presented as the percentage of fluorescent cells.

### Quantification of NAD^+^/NADH and NADP^+^/NADPH

The intracellular NAD^+^ and NADH levels were measured using the NAD^+^/NADH Assay Kit (Abcam, ab65348). The intracellular NADP^+^ and NADPH levels were measured using the NADP^+^/NADPH Assay Kit (Abcam, ab65349). Both kits were used according to the manufacturer’s protocols, and the NAD^+^, NADH, NADP^+^, and NADPH concentrations were determined colorimetrically based on absorbance at 565 nm.

### Quantification of glutathione and glutathione peroxidase activity

Total (GS), oxidized (GSSG) and reduced (GSH) glutathione concentrations were measured using the GSH/GSSG Ratio Detection Assay Kit (Abcam, ab138881) and a fluorescence microplate reader with excitation and emission wavelengths of 490 and 520 nm, respectively. Glutathione peroxidase (GPx) was determined using the BioVision Glutathione Peroxidase Activity Assay Kit (BioVision, Milpitas, CA). One unit of enzyme activity equaled 1.0 µmol of NADPH oxidized to NADP^+^ per minute at 25 °C based on absorbance at 340 nm.

### Quantification of NADPH oxidase activity

NADPH oxidase activity was measured using a cytochrome c reduction assay, as described previously (Yamaura et al. 2009), with slight modification. Cell protein extracts were diluted to 80 µg/well in DMEM without phenol, after which reduction was performed using 400 µM cytochrome c, 80 µM NADPH, and 10 mM Tiron or vehicle control. After incubation at 37 °C for 30 min, cytochrome c reduction was determined based on absorbance at 530 nm. Activity is expressed as the reduction of cytochrome c per milligram protein per minute at 25 °C.

### RNA–protein coimmunoprecipitation

LncRNA-GAS5 cDNA was generated by reverse transcription and PCR, and the cDNA was ligated into pGEM-T Easy (Promega, Madison, WI, USA) to generate GAS5-pGEM-T. LncRNA-GAS5 was transcribed in vitro and biotin-labeled using Biotin RNA Labeling Mix (Sigma-Aldrich). After treatment with RNase-free DNase I, the lncRNA-GAS was purified using the RNeasy Mini Kit (Qiagen, Hilden, Germany), and folded in RNA structure buffer. Total protein (1 mg) from A375 cells transfected with GAS5-pGEM-T was combined with 50 pmol of biotinylated GAS5 before purification using streptavidin-coated magnetic beads (New England Biolabs, Beverly, MA, USA). Aliquots of pulled-down complexes were subjected to SDS-PAGE, western blotting, and silver staining. Proteins in the predicted size ranges were excised, and analyzed by mass spectrometry in a LTQ XL linear ion trap mass spectrometer (Thermo Fisher Scientific) using the micrOTOF focus II data acquisition software (Bruker Daltonics, Billerica, MA, USA). RNA immunoprecipitation (RIP) was performed using the Magna RIP RNA-Binding Protein Immunoprecipitation Kit (EMD Millipore, Bedford, MA, USA), according to the manufacturer’s instructions using a 1:1000 dilution of anti-G6PD antibody. Aliquots of A375 cell lysate (input) and immunoprecipitate were subjected to SDS-PAGE and western blotting using G6PD antibody. The remaining immunoprecipitate was subjected to proteinase K digestion to purify the coprecipitated RNAs, and the identity of the RNA molecules were determined by paired-end RNA sequencing on the Illumina platform (Illumina-China, Beijing).

### Statistical analysis

All the statistical analysis were performed with SPSS version 21.0 (IBM, Armonk, NY, USA) using data obtained from at least three independent experiments. Data are presented as the mean and standard deviation. Data sets were compared using a Chi-squared analysis or Fisher’s exact test for the demographic and clinicopathological variables and two-tailed Student’s *t* tests for continuous variables. The level of statistical significance set was at *P* < 0.05.

## Results

### GAS5 expression is reduced in MM tumors of patients with advanced disease

Given that differential GAS5 expression was associated with poor clinical outcome and reduced survival in patients with a variety of other types of cancer (Cao et al. 2014; Yin et al. 2014; Zhang et al. 2013a), we examined the level of GAS5 expression in tumor tissue samples from 47 MM patients using qRT-PCR, and compared it to the level of GAS5 expression in adjacent noncancerous tissues from the same patient. We found that 76.6% of the MM tumor samples exhibited reduced GAS5 expression, compared to that in adjacent noncancerous tissues (*P* < 0.001), and a significantly greater number of patients with Breslow thickness > 4 cm, TNM stage > II, ulceration, and lymph node metastasis exhibited reduced levels of GAS5 in their MM tissues (*P* < 0.001 for all). The level of GAS5 did not differ significantly based on sex or age (*P* > 0.05; Table 1). These results showed that lncRNA GAS5 expression was significantly lower in MM patients with severe clinicopathological features (*P* > 0.05; Table 1).

**Table 1:** High and low lncRNA GAS5 expression in malignant melanoma in the study population (*n* = 47) and stratified according to demographic and clinical variables

Variable/group	GAS5 expression^a^		*P* value^b^
	Low	High	
Study sample (*n*, [%])	36 (76.6)	11 (23.4)	
Age (years)			0.390
≤ 60	21	8	
> 60	15	3	
Sex			0.117
Men	16	2	
Women	20	9	
Breslow thickness			< 0.001
≤ 4 mm	6	9	
> 4 mm	30	2	
Ulceration			< 0.001
Without	7	11	
With	29	0	
Lymph node metastasis			< 0.001
Negative	8	10	
Positive	28	1	
TNM stage			< 0.001
I	2	8	
II/III/IV	34	3	

^a^Relative GAS5 mRNA expression (normalized to level of GAPDH mRNA) compared to relative GAS5 mRNA expression in adjacent noncancerous tissues determined by qRT-PCR

^b^Based on chi-squared analysis or Fisher’s exact test for the demographic and clinicopathological variables

### GAS5 expression is inversely related to cell viability in MM cell lines

**Fig. 1:**
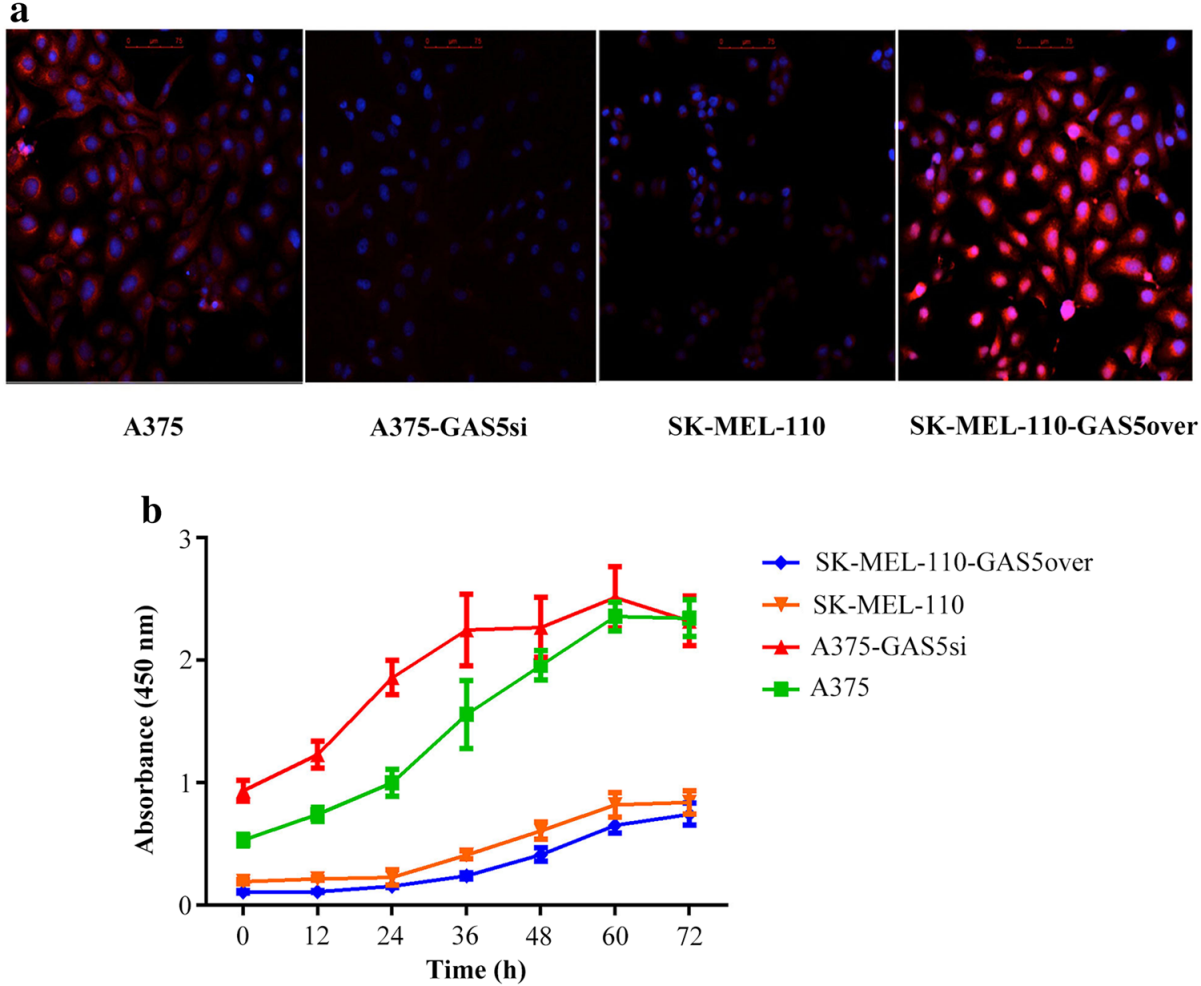
FISH analysis of GAS5 expression and MTT assay assessment of the effect of GAS5 expression level on viability of malignant melanoma cells. **a** A375, A375-GAS5si, SK-Mel-110, and SK-Mel-110-GAS5over cells were probed with a TAMRA-labeled antisense GAS5 oligonucleotide (red), and nuclei were counterstained with DAPI (blue). Confocal microscopy images were merged to show the combined fluorescence. Scale bars, 75 µm. **b** The viability of A375, A375-GAS5si, SK-Mel-110, and SK-Mel-110-GAS5over cells at 12, 24, 36, 48, and 72 h was evaluated by MTT assay. Data points represent mean absorbance at 450 nm from three independent experiments. Error bars represent standard deviation

In our previous study, we showed that the level of GAS5 expression in A375 cells was approximately 33-fold higher than that in SK-Mel-110 cells (Chen et al. 2016b). We also reported that the level of GAS5 expression in SK-Mel-110-GAS5over cells was approximately 120-fold higher than that in SK-Mel-110 cells, whereas the level of GAS5 expression in A375-GAS5si cells was approximately 4.6-fold lower than that in A375 cells (Chen et al. 2016b). This trend was visible in the FISH analysis of GAS5 expression (Fig. 1a). The lower levels of GAS5 expression in SK-Mel-110 and A375-GAS5si cells were visible as scant red fluorescence, whereas the levels of expression in A375 and SK-Mel-110-GAS5over cells were much greater, with the latter exhibiting the highest level of red fluorescence. The results of the MTT assays showed that the siRNA-mediated knockdown of the GAS5 in A375-GAS5si cells resulted in increased viability at 12, 24, and 36 h, compared to that of A375 cells (Fig. 1b). By contrast, overexpression of GAS5 in SK-Mel-110-GAS5over cells resulted in reduced viability, compared to that of SK-Mel-110 cells (Fig. 1b). These results suggested that reduced GAS5 expression contributes to increased MM cell viability.

### GAS5 induces cell cycle arrest at G0/G1 in MM cells

**Fig. 2:**
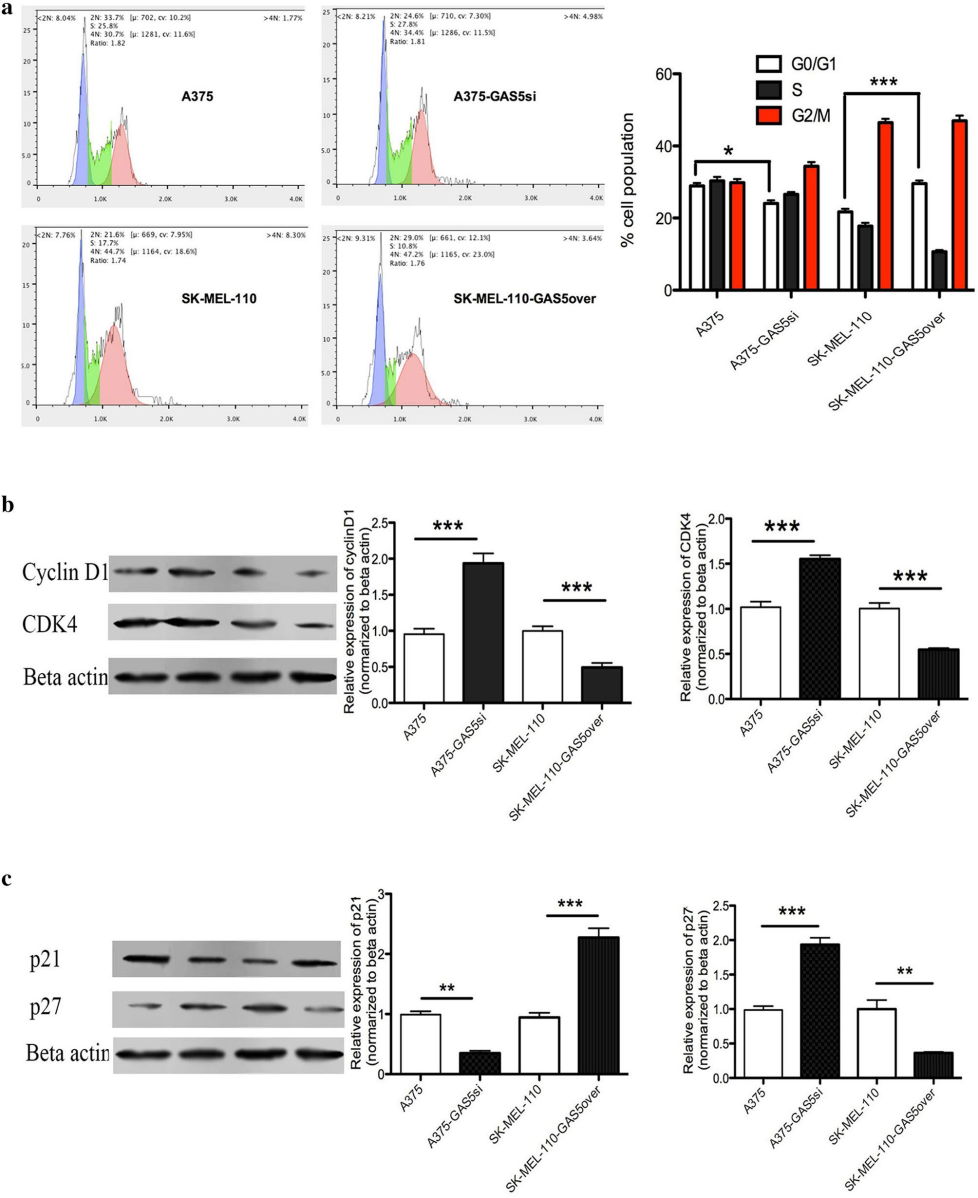
Flow cytometry and western blotting analysis of the effect of GAS5 expression on cell cycle regulation in malignant melanoma cells. **a** Flow cytometry results of propidium iodide stained A375, A375-GAS5si, SK-Mel-110, and SK-Mel-110-GAS5over cells showing the percentage of the cells in G0/G1 (blue), S (green), and G2/M (red) phases, and the same data presented in histogram format. **b, c** Western blot analysis of A375, A375-GAS5si, SK-Mel-110, and SK-Mel-110-GAS5over cell lysates evaluating **b** Cyclin D1 and CDK4 protein expression and **c** p21 and p27 protein expression. Results are expressed in histogram format as the ratio of target band density to that of the β-actin loading control (mean ± standard deviation) for three independent experiments (*P < 0.05, **P < 0.01, ***P < 0.001)

To determine how GAS5 expression influenced the viability of the MM cell lines, we examined differences in cell cycle distribution between A375, A375-GAS5si, SK-Mel-110, and SK-Mel-110-GAS5over cells using a flow cytometry analysis of PI-stained cells. Distinct differences were observed between the fractions of cells in the G0/G1, S, and G2/M phases for the four cell lines examined (Fig. 2a). The percentage of A375 cells in the G0/G1 phase (28.91 ± 1.03%) was significantly greater, compared to the percentage of A375-GAS5si cells in the G0/G1 phase (24.07 ± 2.18%, *P* < 0.05; Fig. 2a), and the percentage of SK-Mel-110-GAS5over cells in the G0/G1 phase (29.56 ± 1.27%) was significantly greater, compared to the percentage of SK-Mel-110 cells in the G0/G1 phase (21.72 ± 0.39%, *P* < 0.001; Fig. 2a). These results suggested that knockdown of GAS5 expression caused more A375-GAS5si cells to progress past the G0/G1 phase of the cell cycle, compared to that observed in A375 cells, and that the increase in GAS5 expression caused more SK-Mel-110-GAS5over cells to remain in the G0/G1 phase, compared to that observed in SK-Mel-110 cells.

Given the effect of GAS5 expression on cell cycle distribution, we examined the levels of cell cycle-related proteins by western blotting. The levels of Cyclin D1, CDK4, and p27 proteins in A375-GAS5si cells were significantly higher than those in A375 cells (*P* < 0.001 for all; Fig. 2b, c), whereas the level of p21 protein in A375-GAS5si cells was significantly lower than that in A375 cells (*P* < 0.01; Fig. 2c). The levels of the Cyclin D1, CDK4, and p27 proteins in SK-Mel-110 cells were significantly higher than those in SK-Mel-110-GAS5over cells (*P* < 0.001, *P* < 0.001, and *P* < 0.01, respectively; Fig. 2b, c), whereas the level of p21 in SK-Mel-110 cells was significantly lower than that in SK-Mel-110-GAS5over cells (*P* < 0.001; Fig. 2c). Given that Cyclin D1, CDK4, and p27 are markers of active cell division and p21 is a marker of G1/S cell cycle arrest (Liu et al. 2015), the western blotting results suggested that reduced GAS5 expression promoted G1/S progression in A375-GAS5si cells, whereas increased GAS5 expression promoted G1/S arrest in SK-Mel-110-GAS5over cells.

### GAS5 expression inhibits Bcl-2 mediated suppression of apoptosis in MM cells

**Fig. 3:**
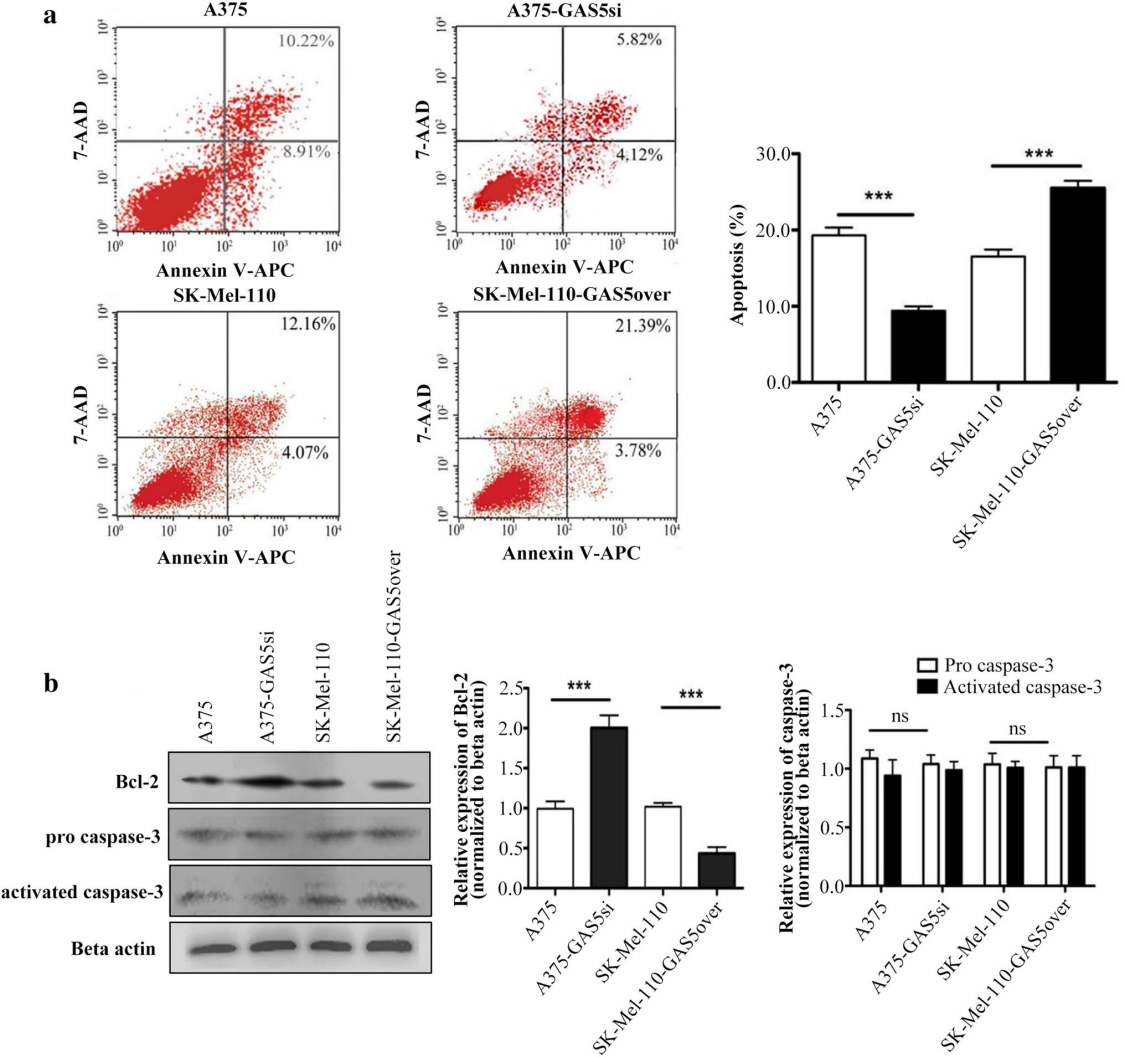
Flow cytometry and western blotting analysis of the effect of GAS5 expression on apoptosis in malignant melanoma cells. **a** Apoptosis status of A375, A375-GAS5si, SK-Mel-110, and SK-Mel-110-GAS5over cells was assessed by qualitative flow cytometry using Annexin V-APC and 7AAD-FITC staining. Necrotic (dead) cells are 7AAD-positive and Annexin V-negative, and are represented in the upper-left quadrant of the plots. Non-viable (late) apoptotic cells are positive for both Annexin V and 7AAD, and are represented in the upper-right quadrant. Viable (early) apoptotic cells are 7AAD-negative and Annexin V-positive, and are represented in the lower-right quadrant. Viable non-apoptotic cells are negative for both Annexin V and 7AAD, and are represented in the lower-left quadrant. **b** Western blot analysis of A375, A375-GAS5si, SK-Mel-110, and SK-Mel-110-GAS5over cell lysates evaluating Bcl-2, pro-Caspase-3, and activated Caspase-3 expression. Results are expressed in histogram format as the ratio of target band density to that of the β-actin loading control (mean ± standard deviation) for three independent experiments (*P < 0.05, **P < 0.01, ***P < 0.001)

To determine whether the effects of GAS5 expression on cell cycle markers influence the regulation of apoptosis in MM cells, we quantified the proportion of cells undergoing apoptosis in populations of A375, A375-GAS5si, SK-Mel-110, and SK-Mel-110-GAS5over cells using Annexin V-APC and 7-AAD double staining and flow cytometry. The knockdown of GAS5 in A375-GAS5si cells reduced the proportion of apoptotic cells by 10.21 ± 1.08% (*P* < 0.001), compared to A375 cells (Fig. 3a). In contrast, overexpression of GAS5 in SK-Mel-110-GAS5over cells increased apoptosis by 6.99 ± 1.30% (*P* < 0.001), compared to SK-Mel-110 cells (Fig. 3a).

To further investigate the effects of GAS5 expression on apoptosis in MM cells, we examined the levels of the apoptotic proteins, Caspase-3 and Bcl-2. Western blotting showed that the GAS5 knockdown in A375-GAS5si cells increased the expression of Bcl-2 by approximately 52.38% (*P* < 0.001), compared to A375 cells (Fig. 3b). In contrast, GAS5 overexpression in SK-Mel-110-GAS5over cells reduced Bcl-2 expression approximately 50% (*P* < 0.001), compared to SK-Mel-110 cells (Fig. 3b). Levels of Caspase-3 in A375-GAS5si and SK-Mel-110-GAS5over cells were similar to those in A375 and SK-Mel-110 cells (*P* > 0.05; Fig. 3b). These flow cytometry and western blotting results suggested that GAS5 expression inhibits Bcl-2 mediated suppression of apoptosis in the MM cell lines.

### GAS5 expression alters ROS level and redox balance in MM cells

**Fig. 4:**
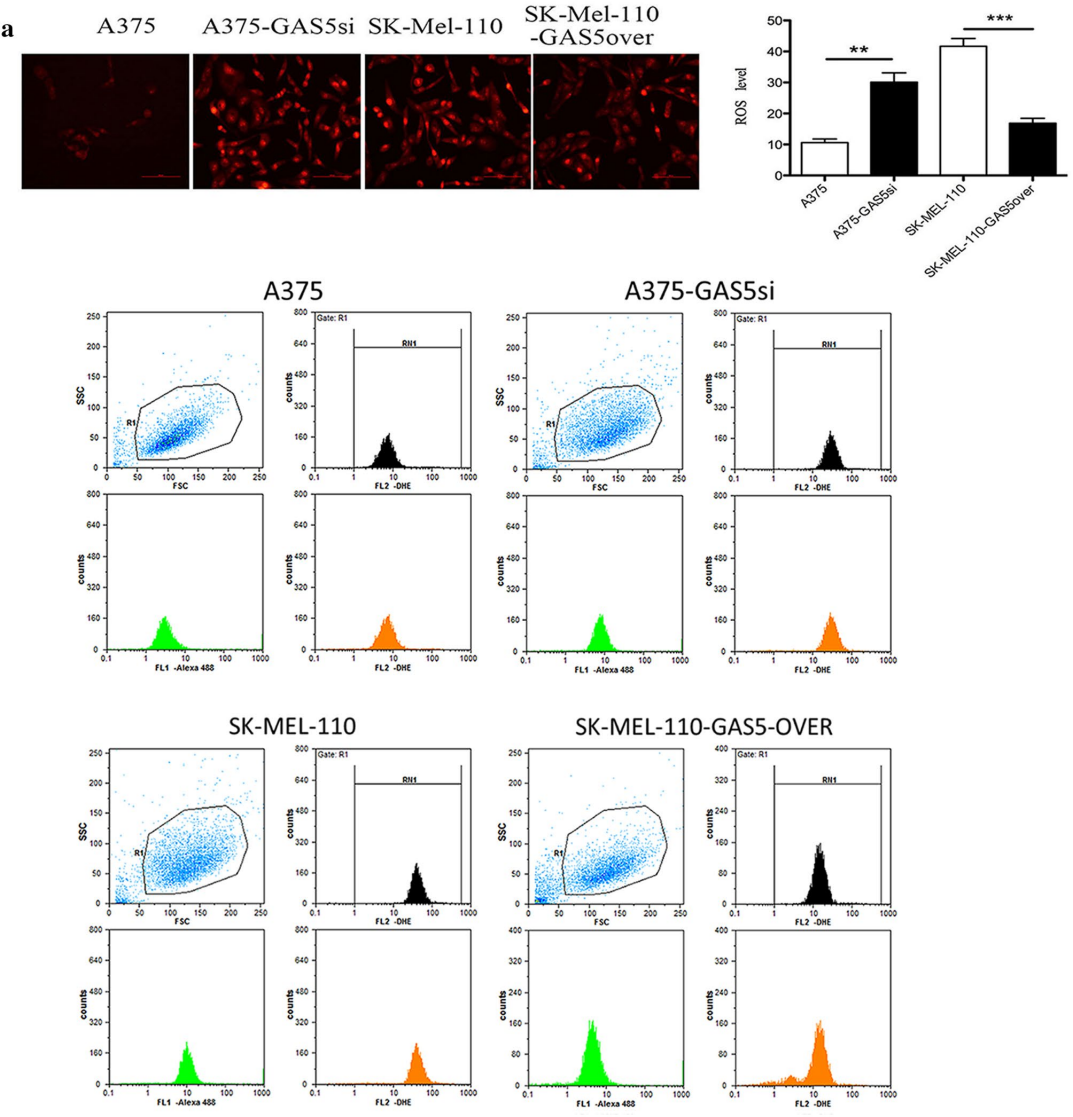

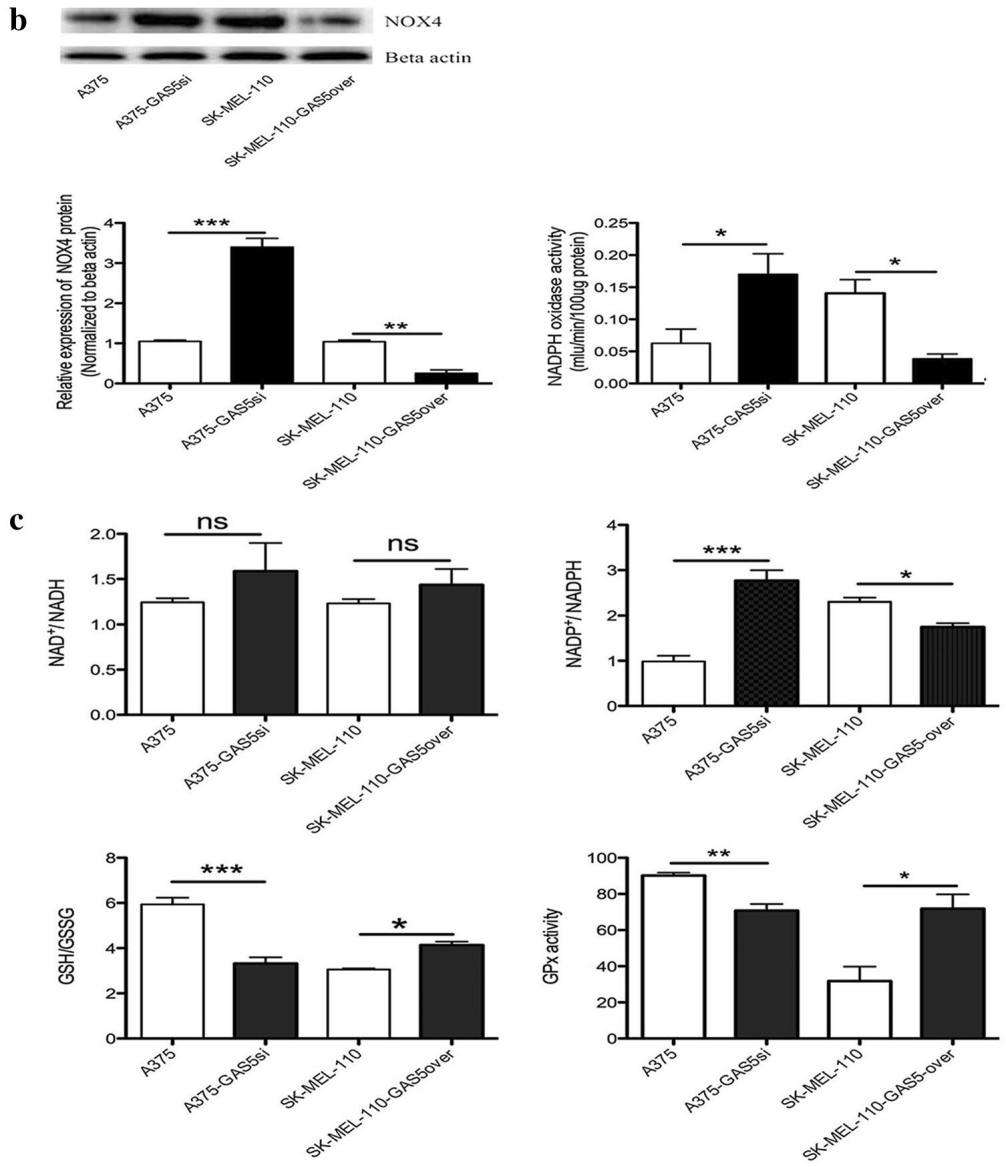
Quantification of reactive oxygen species (ROS) in malignant melanoma cells. **a** Intracellular ROS in A375, A375-GAS5si, SK-Mel-110, and SK-Mel-110-GAS5over cells were measured in vivo based on dihydroethidium (DHE) fluorescence and DAPI staining. (**a**, Upper panels) Images were recorded using fluorescence microscopy before subjecting the cells to fluorescent cell sorting for quantitative analysis. (**a**, Lower panels) Flow cytometry results are presented as the mean fluorescence intensity (MFI) of DHE, and (**a**, right) data are presented in histogram format as mean ± standard deviation from three independent experiments (*P < 0.05, **P < 0.01, ***P < 0.001 vs control cells without DHE). **b, c** A375, A375-GAS5si, SK-Mel-110, and SK-Mel-110-GAS5over cell lysates were subjected to (**b**, center) western blot analysis to measure NOX4 protein expression with western blotting results (**b**, left) expressed as the ratio of target band density to that of the β-actin loading control; (**b**, right) analysis of NADPH oxidase activity with results expressed as the reduction of cytochrome c per milligram protein per minute at 25 °C; and analyses of (**c**, upper left) NAD^+^/NADH ratio, (**c**, upper right) NADP^+^/NADPH ratio, (**c**, lower left) GSH/GSSG ratio, and (**c**, lower right) glutathione peroxidase activity. Data are presented as mean ± standard deviation from three independent experiments (*P < 0.05, **P < 0.01, ***P < 0.001)

Given that ROS levels influence the regulation of apoptosis (Cesi et al. 2017; Conklin 2004), we examined intracellular superoxide anion (O2∸) levels in vivo using DHE fluorescent staining and flow cytometry. In A375-GAS5si cells, the O2∸ level was 62.67 ± 7.22% greater than that in A375 cells (*P* < 0.01; Fig. 4a). In SK-Mel-110-GAS5over cells, the O2∸ level was 68.75 ± 5.64% lower than that in SK-Mel-110 cells (*P* < 0.001; Fig. 4a). These results suggested that GAS5 expression was inversely proportional to the intracellular ROS level in the MM cell lines.

Based on the results of the flow cytometry analysis of ROS levels, we investigated whether the GAS5 induced changes in ROS were mediated by NOX4. As a downstream effector of Akt-mediated signaling, NOX4 catalyzes the reduction of O_2_ to O2∸ and its metabolite, hydrogen peroxide, and contributes to G2/M cell cycle progression in MM cells (Yamaura et al. 2009). Western blotting showed that NOX4 protein expression in A375-GAS5si cells was approximately 2.2-fold higher (*P* < 0.001) than that in A375 cells (Fig. 4b). By contrast, NOX4 expression in SK-Mel-110-GAS5over cells was approximately 2.9-fold lower (*P* < 0.01) than that in SK-Mel-110 cells (Fig. 4b). NADPH oxidase activity in A375-GAS5si cells was approximately 1.6-fold higher than that in A375 cells, whereas NADPH oxidase activity in SK-Mel-110-GAS5over cells was approximately 2.2-fold lower than that of SK-Mel-110 cells (*P* < 0.05 for both; Fig. 4b).

To further investigate the effects of GAS5 expression on ROS homeostasis in MM cells, we examined differences in GPx activity and the NAD^+^/NADH, GSH/GSSG, and NADP^+^/NADPH ratios between cell lines. In A375-GAS5si cells, the NADP^+^/NADPH ratio was 179.00 ± 26.0% higher (*P* < 0.001), while the GSH/GSSG ratio and GPx activity were 43.84 ± 4.02% (*P* < 0.001) and 21.58 ± 3.79% (*P* < 0.01) lower, respectively, compared with those in A375 cells (Fig. 4c). In SK-Mel-110-GAS5over cells, the NADP^+^/NADPH ratio was 24.17 ± 1.37% lower, while the GSH/GSSG ratio and GPx activity were 35.43 ± 3.52% and 129.00 ± 14.0% higher, respectively, compared with those in SK-Mel-110 cells (*P* < 0.05 for all; Fig. 4c). The NAD^+^/NADH ratios in A375-GAS5si and A375 cells were similar (*P* > 0.05), as were those in SK-Mel-110-GAS5over and SK-Mel-110 cells (*P* > 0.05). These results suggested that increased NOX4 expression and NOX activity in A375-GAS5si cells increased levels of NADP^+^ and oxidized glutathiones.

### GAS5 influences the effect of ROS level on Bcl-2 and Cyclin D1 expression

**Fig. 5:**
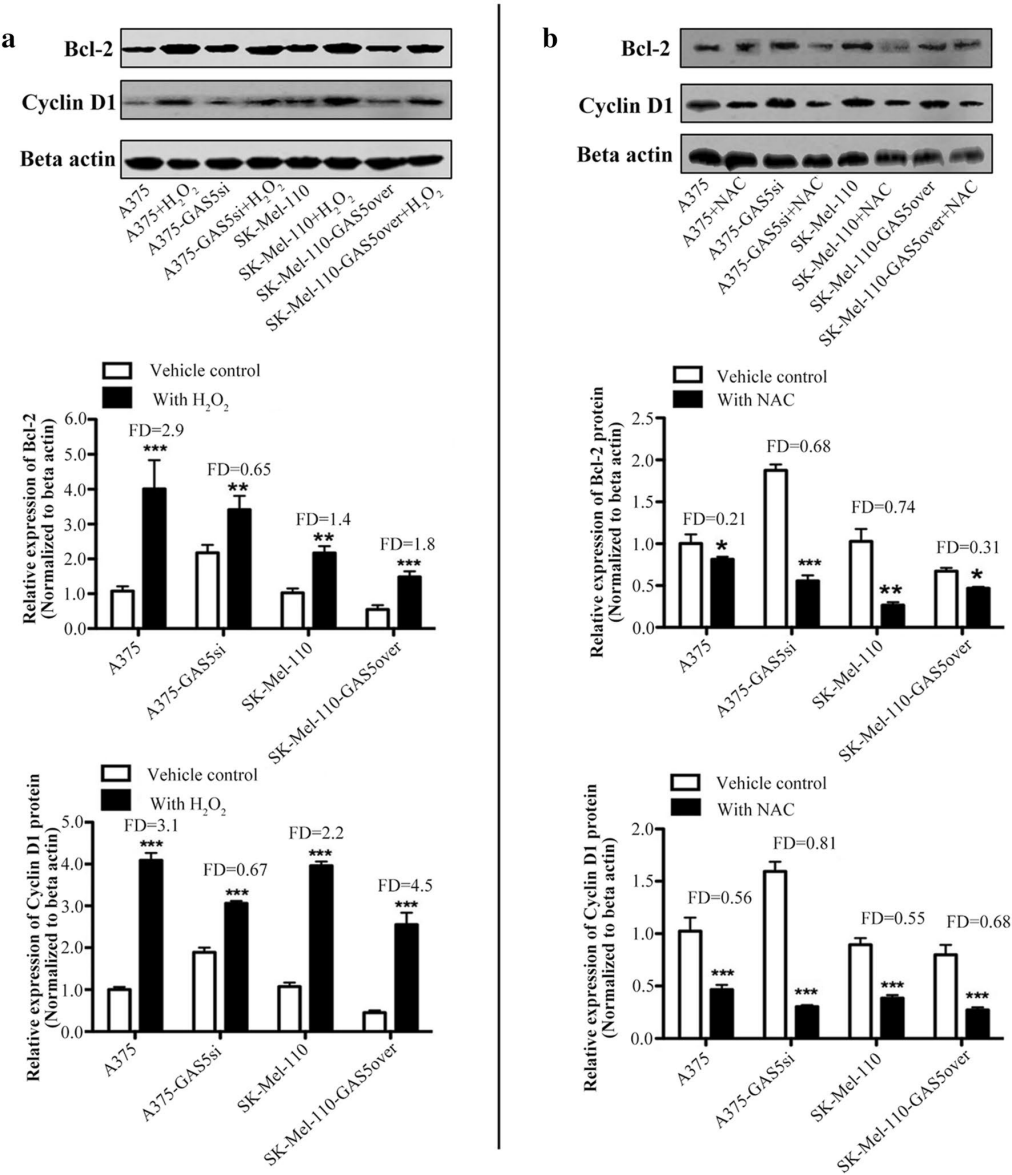
Western blot analysis of the effect GAS5 expression on ROS-induced changes in Bcl-2 and Cyclin D1 expression in melanoma cells. A375, A375-GAS5si, SK-Mel-110, and SK-Mel-110-GAS5over cells were treated with **a** 50 µM H_2_O_2_ for 45 min or **b** 100 µM *N*-acetyl-cysteine (NAC) for 45 min, after which cell lysates were prepared and subjected to western blotting to detect (top) Bcl-2 and Cyclin D1 protein expression. Western blotting results for (middle) Bcl-2 and (bottom) Cyclin D1 are expressed in histogram format as the ratio of target band density to that of the β-actin loading control (mean ± standard deviation) for three independent experiments (*P < 0.05, **P < 0.01, ***P < 0.001). Results were normalized based on the level of expression in A375 cells. Fold difference (FD) was calculated for each cell line by comparing the protein level in the treatment group to the level in the vehicle control group

To investigate whether GAS5 expression influences the effects of ROS levels on cell cycle and apoptosis related proteins, we treated A375, A375-GAS5si, SK-Mel-110, and SK-Mel-110-GAS5over cells with NAC or H_2_O_2_ prior to western blotting. Treatment with H_2_O_2_ significantly increased Bcl-2 expression in A375, A375-GAS5si, SK-Mel-110, and SK-Mel-110-GAS5over cells, resulting in 2.9-, 0.65-, 1.4-, and 1.8-fold increases, respectively (Fig. 5a). Cyclin D1 expression also increased significantly in A375, A375-GAS5si, SK-Mel-110, and SK-Mel-110-GAS5over cells treated with H_2_O_2_, resulting in 3.1, 0.67, 2.2, and 4.5-fold increases, respectively (Fig. 5a). Treatment with NAC significantly reduced Bcl-2 expression in A375, A375-GAS5si, SK-Mel-110, and SK-Mel-110-GAS5over cells, resulting in 0.21-, 0.68-, 0.74-, and 0.31-fold decreases, respectively (Fig. 5b). Cyclin D1 expression was also reduced significantly in A375, A375-GAS5si, SK-Mel-110, and SK-Mel-110-GAS5over cells treated with NAC, resulting in 0.56-, 0.81-, 0.55-, and 0.68-fold decreases, respectively (Fig. 5b). These results suggested that changes in Bcl-2 and Cyclin D1 expression induced by altered redox balance are influenced by the level of GAS5 expression.

### GAS5 regulates redox balance through G6PD

**Fig. 6:**
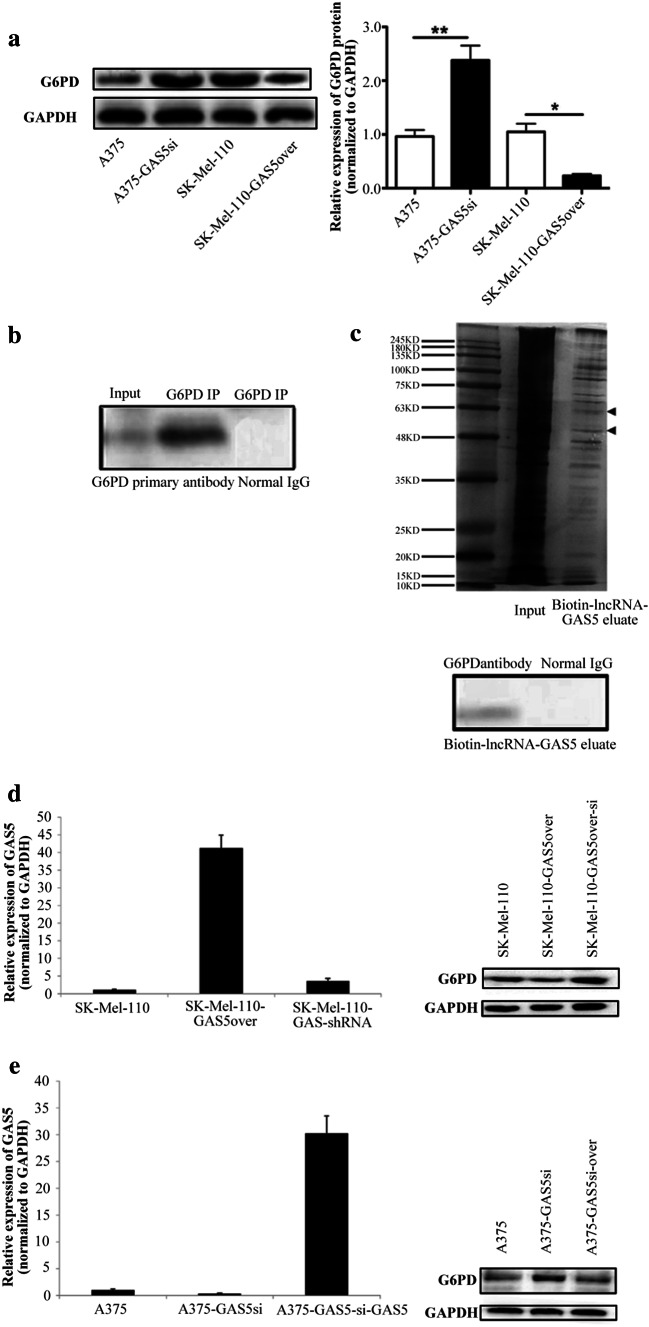
Western blot analysis of G6PD expression in melanoma cells, and detection of physical interaction between GAS5 and G6PD protein using RNA immunoprecipitation (RIP) and RNA pull down assay. **a** A375, A375-GAS5si, SK-Mel-110, and SK-Mel-110-GAS5over cell lysates were subjected to (**a**, left) western blot analysis to measure G6PD protein expression with western blotting results, and (**a**, right) the results are expressed in histogram format as the ratio of target band density to that of the GAPDH loading control (mean ± standard deviation) for three independent experiments (*P < 0.05, **P < 0.01, ***P < 0.001). **b** A375 cell lysate was subjected to RIP using a 1:1000 dilution of anti-G6PD antibody. Aliquots of the A375 cell lysate (input) and immunoprecipitate were subjected to SDS-PAGE and western blotting using anti-G6PD antibody or normal IgG (control). The remaining immunoprecipitate was subjected to proteinase K digestion to purify the coprecipitated RNAs, and the RNA molecules were identified by RNA seq analysis. **c** RNA pull down assay of A375 cell lysate supplemented with 50 pmol biotinylated GAS5. RNA binding complexes were isolated using streptavidin-coated magnetic beads, and the biotin-lncRNA-GAS5 eluate was subjected to SDS-PAGE. Gels were subjected to (**c**, upper) silver staining and (**c**, lower) western blotting using anti-G6PD antibody or normal IgG (control). (**d**, Left) SK-Mel-110-GAS5over cells were transfected with pSIREN-RetroQ-ZsGreen1-GAS5shRNA for GAS5 knock down to generate SK-Mel-110-GAS5over-si cells, and (**d**, right) G6PD expression was evaluated by western blotting based on comparisons to G6PD expression in SK-Mel-110 and SK-Mel-110-GAS5over cells (GAPDH as loading control). (**e**, Left) GAS5 was overexpressed in the A375-GAS5si cells using a lentivirus expression system to generate A375-GAS5si-over cells, and (**e**, right) G6PD expression was evaluated by western blotting based on comparisons to G6PD expression in A375 and A375-GAS5si cells (GAPDH as loading control)

A previous studied showed that ROS levels in A375 cells increase in response to the increased expression of G6PD (Cai et al. 2015). Therefore, we investigated whether GAS5 influenced the expression of G6PD by western blotting. For western blot quantification, the level of G6PD expression was normalized to that of GAPDH because GAPDH expression is not affected by the NAD^+^/NADH ratio. Western blotting showed that level of G6PD protein in A375-GAS5si cells was significantly higher than that in A375 cells (*P* < 0.01), whereas the level of G6PD protein in SK-Mel-110-GAS5over cells was significantly lower than that in SK-Mel-110 cells (*P* < 0.05; Fig. 6a). These results suggested that the reduced NADP^+^/NADPH ratio in A375 cells (Fig. 4c) was due to a G6PD-induced increase in NADPH, and that the increased expression of NOX4 in A375-GAS5si cells resulted in greater conversion of NADPH to NADP^+^, thereby increasing the NADP^+^/NADPH ratio and ROS level (Fig. 4a). This shift in redox balance as the result of GAS5 knockdown is consistent with the lower NADP^+^/NADPH ratio and ROS level due to reduced G6PD and NOX4 expression in cells overexpressing GAS5.

Given the effects of GAS5 of the expression of G6PD and ROS levels, we examined possible interactions between GAS5 and the G6PD protein using RIP and RNA pull-down assays. In the RIP assays, western blotting confirmed the presence of G6PD in the immunoprecipitate from A375 cell lysates (Fig. 6b). Results of the RNA seq analysis of the G6PD co-precipitates identified significant GAS5 enrichment (FPKM = 2410, Supplementary Data 1). In the RNA pull-down assays, SDS-PAGE of complexes containing biotinylated GAS5 RNA produced two clear bands between 48 and 63 kDa (Fig. 6c) that were similar in size to the G6PD protein (59 kDa), and western blotting identified a 59-kDa band (Fig. 6c). However, mass spectrometry analysis did not identify G6PD protein in the bands excised from gels (data not shown). These results showed that, in MM cells, a physical interaction occurs between GAS5 and the G6PD protein, and suggested that the role of G6PD in redox balance is mediated by its interaction with GAS5.

In rescue experiments, we knocked down GAS5 expression in SK-Mel-110-GAS5over cells, and western blotting showed that the level of G6PD expression was clearly reduced, compared to G6PD expression in both SK-Mel-110 and SK-Mel-110-GAS5over cells (Fig. 6d). We also overexpressed wild-type GAS5 in A375-GAS5si cells at a very high level using a lentivirus vector, and western blotting showed that G6PD expression in A375-GAS5si-over cells was intermediate to that in A375 and A375-GAS5si cells. The results of these experiments further suggest that GAS5 influences redox balance in MM cells by altering the level G6PD expression (Fig. 6e).

## Discussion

In our current study, we found that GAS5 expression was significantly lower in tumors from patients with advanced disease (76.6%, *P* < 0.001), relative to that in adjacent noncancerous tissues, as evidenced by larger tumor size, higher TNM stage, and higher incidences of ulceration and metastasis, compared to those of patients whose tumors expressed levels of GAS5 that were higher than those in their control tissues (Table 1). MTT assays showed that the level of GAS5 expression was inversely related to cell viability (Fig. 1b). Flow cytometry and western blotting showed that reduced GAS5 expression in A375-GAS5si cells induced G1/S progression by increasing Cyclin D1, CDK4, and p27 expression (Fig. 2), and inducing Bcl-2 mediated suppression of apoptosis, without affecting Caspase-3 levels (Fig. 3). Our analysis of the effects of GAS5 on redox balance in MM cell lines showed that GAS5 expression influenced changes in Bcl-2 and Cyclin D1 expression in response to altered redox balance (Fig. 5), and that GAS5 knockdown increased levels of NADP^+^, O2∸, and oxidized glutathiones (Fig. 4a, c) by increasing NOX4 expression, NADPH oxidase activity (Fig. 4b), and G6PD expression (Fig. 6a) through a mechanism that involved a physical interaction between GAS5 and the G6PD protein (Fig. 6b).

Previous studies have shown that GAS5 functions as a tumor suppressor by promoting apoptosis in breast cancer (Pickard and Williams 2014, 2016) and both apoptosis and cell cycle arrest in neuroblastoma (Mazar et al. 2017). In another study, Cai et al. (2015) showed that the expression of G6PD and/or NOX4 increased A375 cell proliferation through c-SRC and SHP2 regulated STAT3-mediated signaling pathways. The findings of these previous studies are consistent with our observations that GAS5 knockdown increased A375-GAS5si cell viability, and increased G6PD and NOX4 expression. Cai et al. (2015) also reported that silencing G6PD and NOX4 expression in A375 cells reduced Cyclin D1 and CDK4 expression while increasing p53 and p21 expression, which are consistent with our observations that A375-GAS5si cells had higher levels of CDK4 and Cyclin D1 and a lower level of p21, compared to those in A375 cells. Our observation that GAS5 knockdown-induced G1/S progression is supported in part by a recent report by Wang et al. (2017), who showed that a 2-*O*-methylmagnolol-induced increase in GAS5 expression increased apoptosis in A375 cells. Wang et al. (2017) also reported a high rate of reduced GAS5 expression in MM tumor tissues, which is consistent with our observation that a significant portion of the clinical MM samples exhibited reduced GAS5 levels. Therefore, our findings and those of previous studies suggest that GAS5 knockdown-induced G1/S progression occurs via STAT3-mediated signaling pathways, and further suggest that reduced GAS5 expression contributes to tumor progression in MM patients.

Although it is becoming increasingly clear that redox balance and ROS homeostasis are important factors in MM tumorigenesis, the specific mechanisms by which these processes contribute to MM progression remain largely unclear (Wittgen and van Kempen 2007). Resistance to BRAF inhibitors in MM patients who have activating mutations in the RAS/RAF/MEK/ERK pathway is most often accompanied by an increase in ROS levels (Cesi et al. 2017). Multiple studies have reported significantly elevated NOX1 and/or NOX4 expression in MM tumors of different stages (Liu-Smith et al. 2014; Yamaura et al. 2009). The expression of NOX4 is thought to contribute to G1/S progression in MM cells based on the reduction of ROS and inactivation of CDK1 in response to NOX4 knockdown in different MM cell lines (Cai et al. 2015; Yamaura et al. 2009). G6PD knockdown has been shown to reduce ROS levels in A375 cells (Cai et al. 2015). Our report of increased ROS, NOX4, and G6PD levels in A375-GAS5si cells is consistent with the findings of these previous studies.

Spencer et al. (2011) showed that both G6PD and NOX4 contribute to O2∸ production in hepatocytes, which is consistent with our finding that the O2∸ level in A375-GAS5si cells was higher than that in A375 cells (Fig. 4a). Hu et al. (2013) showed that the loss of G6PD expression reduced A375-G6PDΔ cell proliferation in a mouse xenograft model of MM, which is consistent with our observations that the increase in G6PD expression in A375-GAS5si cells (Fig. 6a) coincided with reduced apoptosis (Fig. 3a). Hu et al. (2013) also reported that Bcl-2 expression was significantly reduced in A375-G6PDΔ cells while the expression of Fas was significantly increased. Therefore, our finding that Bcl-2 expression was higher in A375-GAS5si cells than in A375 cells suggests that the anti-apoptotic effect of GAS5 knockdown was mediated by G6PD.

Our findings are subject to certain limitations. Our analysis of GAS5 levels in MM tumors included samples from only 47 patients. Other investigators have also reported low GAS5 expression in MM tumors, relative to adjacent control tissues (Wang et al. 2017). An analysis of GAS5 expression in a larger patient population is required to determine whether reduced GAS5 expression correlates with MM tumorigenesis and advanced disease. Our inability to identify G6PD in the mass spectrometry analysis of co-immunoprecipitates from A375-GAS5si cells might be viewed as a confounding factor in our overall analysis, but it is possible that the level of G6PD protein in the sample was below the detectable limit of the instrument. In addition, we did not examine the levels of CDK1, p53, or activated STAT3 in A375-GAS5si cells, nor did we examine the levels of NOX4, Bcl-2, or G6PD in the MM tumor tissues. Additional experiments are required to determine the signaling pathways that contribute to increased proliferation of A375-GAS5si cells, and to investigate the mechanisms by which GAS5 knockdown elevates O2∸ and oxidized glutathione levels. Further study is also required to examine whether the effects of reduced GAS5 expression in the tumors of MM patients mirrors the induction of G1/S cell cycle progression in A375-GAS5si cells. How the binding of G6PD to GAS5 is mechanistically linked to the regulation of G6PD, NOX4, Bcl-2, Cyclin D1, CDK4, p27, and p21 expression is unclear. It is possible that GAS5 binding influences G6PD enzymatic activity or alters protein–protein interactions between G6PD and cell cycle or apoptosis related proteins, such as pro-apoptotic p53, which negatively regulates G6PD activity in oxidative metabolism (Jiang et al. 2011).

## Conclusions

Our findings show GAS5 contributes to regulation of the apoptosis, cell cycle, homeostasis of reactive oxygen species, and redox balance in MM cells, and suggest that reduced GAS5 expression contributes to disease progression in MM patients. Our ongoing investigation of the role of GAS5-G6PD binding in MM cell proliferation and apoptosis will provide important insights into the functions of lncRNAs. Determining whether the mechanism underlying increased O2∸ levels in A375-GAS5si cells is similar to that in BRAF-inhibitor-resistant MM tumors is of great importance. Future studies of the effects of GAS5 expression on O2∸ production and the regulation of ROS scavengers might contribute to a better understanding of altered ROS levels in drug-resistant MM.

## Electronic supplementary material

Below is the link to the electronic supplementary material.

Supplementary material 1 (XLS 32 KB)
